# Impact of COVID-19 pandemic on the residency programs of the country: A multicentre study

**DOI:** 10.12669/pjms.37.2.3496

**Published:** 2021

**Authors:** Laima Alam, Mafaza Alam, Syed Kumail Hasan Kazmi, Syed Ashoor Hasan Kazmi

**Affiliations:** 1 Laima Alam, FCPS. Junior Consultant Gastroenterology, Bahria Town International Hospital, Phase VIII, Rawalpindi, Pakistan; 2 Mafaza Alam Registrar Operative Dentistry, Armed Forces Institute of Dentistry, Rawalpindi, Pakistan; 3 Syed Kumail Hasan Kazmi, FCPS, Fellow Gastroenterology, Pak Emirates Military Hospital, Rawalpindi, Pakistan; 4 Syed Ashoor Hasan Kazmi, Registrar Trauma and Orthopaedics, University Hospital Birmingham, United Kingdom

**Keywords:** COVID-19, Pandemic, Residents, Survey, Training

## Abstract

**Objective:**

To provide an insight on the disruption of multiple facets of residency programs in a multi-centre study.

**Methods:**

This cross-sectional survey was carried out by enrolling the available residents from three teaching hospitals of the country by sending a questionnaire through email. The questionnaire comprised of three parts; 1) basic demographics, 2) effect on multiple facets of training and 3) the use of smart learning with the support provided by the hospitals. Data collection was started during the first week of June 2020 after acquiring ethical approval from the concerned department and the total duration of the study was one month. Data was analysed using SPSS v. 19.0.

**Results:**

A hundred-and-five completed responses were obtained with a response rate of 42%. Fifty-nine percent of the participants were female residents. Majority of the residents (69%) belonged to the age group 25-30 years. Fourth year residents (38%) showed maximum participation and the mean number of work days per month were 22±5.4. All of the aspects of training suffered complete or severe reduction except for the multi-disciplinary team (MDT) meetings, elective rotations and e-log book entries. Sixty seven to sixty-nine percent of the residents felt complete clinical, educational and psychological desertion in their departments, 59% used telemedicine and 90% reported non-availability of smart learning facilities.

**Conclusion:**

Overall, our study confirmed that the COVID-19 pandemic has substantially affected the clinical skills, teaching and personal growth of many trainees. There is a decrease in exposure to almost all of the aspects of training with no alternative in the form of smart learning provided to many. Clinical, educational and psychological support, although an extremely important part of healthcare staffing and management, has been largely neglected as well.

## INTRODUCTION

The number of active cases and deaths secondary to COVID-19 are rising rapidly following a steep ascending curve.^1^ Social distancing and implementation of safe practices have impacted many post-graduate medical training programs across the country, resulting in severe disruption of clinical and educational skill procurement.^2^ It has become an international necessity to balance the mandatory social distancing required for slowing the disease spread while catering to the educational and clinical needs of the trainee doctors who would be running the wards and clinics on their own in near future after accreditation. Also, maintaining a steady supply of workforce in these unprecedented conditions for providing clinical care to the masses, flexibility in the current health care guidelines, adjustments in trainee scheduling and flow, re-evaluation of resident duties and prospects, and the need for frequent assessments in a safe environment are needed.^3^

The re-percussions of the unprecedented challenges are being felt almost by all specialties and fellowship programs of the medical field.^4^ Many of the residents belonging to diverse specialties have been redeployed to cater COVID-19 patients and wards, causing cancellation of elective procedures (normally done by residents) and elimination of Out Patient visits which were the main sources of learning for the residents.^5^ The balance between clinical work, research, educational aspect of training and personal growth and development has been disrupted severely.^6^

Many of the residents are and will be side-lined by self-isolation, hospital admissions and possible deaths while performing their front-line duties during these chaotic times.^4^ This pandemic will change the educational and clinical experience of many fellows across the globe.^7^ The constant pressure to acquire clinical skills, provide care, stay healthy and to prevent the community spread of the virus by not becoming a vector has been emotionally and psychologically crippling for many doctors and residents.^8^

We present a cross-sectional survey, one of its own kind, locally and internationally, involving residents from many different specialties of three large teaching hospitals of the country to gauge the possible impact of the pandemic on their training routines.

## METHODS

This cross-sectional survey was carried out enrolling the available residents from three teaching hospitals namely, Pak Emirates Military Hospital Rawalpindi, Fauji Foundation Hospital Rawalpindi and Armed Forces Institute of Dentistry Rawalpindi by sending a questionnaire through email, using convenience sampling. Data collection was started during the first week of June 2020 after acquiring ethical approval from the concerned department (A/28/07/EC/121 dated 01-06-2020) and the total duration of the study was one month. The survey was created by an online survey generator.^9^ A reminder was given to the residents after one week of no response and the candidates were dropped who failed to respond after another three days.

The questionnaire was developed by LA and MA after relevant literature review. It was reviewed by two medical education experts for content validity. The survey was piloted among 15 post-graduate residents before putting it to test. The questionnaire was divided in to three parts. The first encompassed the demographic data including age, gender, specialty of residency, year of residency program and the number of days spent in the hospital per month during the pandemic. The second part comprised of three main aspects of residency programs affected by the current situation; clinical (active and passive learning), teaching (receiving and giving) and personal growth and development, all of which were evaluated using 8 questions each, by taking help from the training module of Royal College of Physicians and Australian Medical Council Limited. The percentage decrease of the residents’ involvement in all three aspects of training was classified as slight for <40% reduction, severe for 40-80% reduction and complete suppression for >80% reduction^10^ (the already defined percentages were used as surrogate metrics to represent the suppressed training activities as the referenced study) than the pre-covid days. The referenced study is a pioneer study and no such study has been carried out keeping in view the multiple facets of post-graduate training. The third part had Yes/No questions regarding the use of telemedicine, use of online assessments and classes, and the support provided from the hospital psychologically and educationally.

The sample size was calculated with margin of error set at 6%, confidence level at 95% and an anticipated frequency (response distribution) of 65% using OpenEpi sample size calculator. Thus a total of 250 surveys were sent out through email to residents of all medical, surgical and allied specialties. Data was statistically described in terms of mean ± SD for continuous data, frequencies and percentages when appropriate. Chi square test and Fisher exact test were used to compare qualitative data. All statistical analyses were performed using SPSS v 19.0. All p values ≤0.05 were considered statistically significant.

## RESULTS

**Fig.1:**
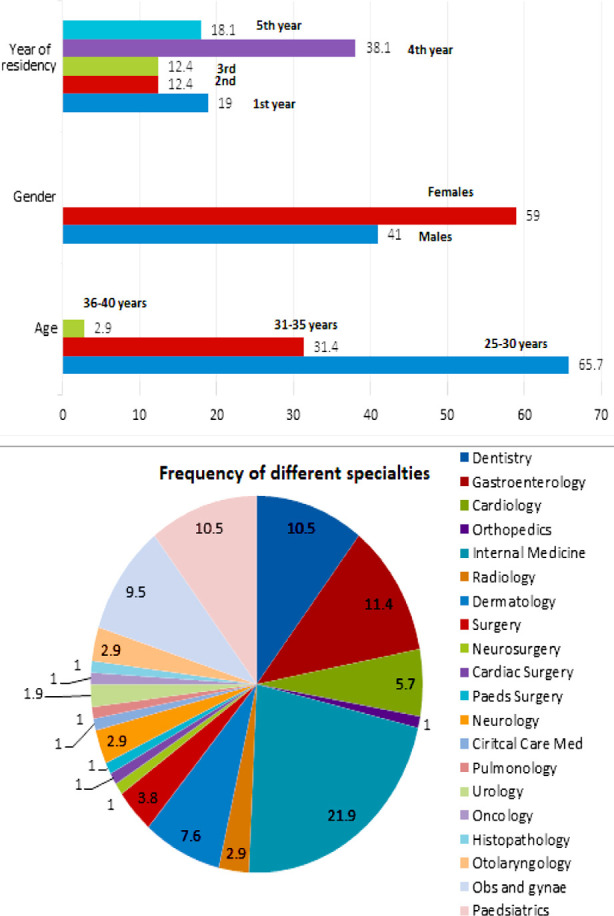
Demographics of the residents enrolled in the study.

Hundred and nine residents filled the survey within the time frame, four responses were incomplete and thus discarded. The response rate was 42%. Female residents were in abundance (59%). Majority of the residents (69%) belonged to the age group 25 to 30 years. Fourth year residents (38%) showed preponderance which happens to be the last year of residency for many specialties (Fig.1). The mean number of work days per month were 22 ± 5.4.

Majority of the residents reported severe to complete reduction in all of the facets of teaching except for the Multi-disciplinary Team meetings (Table-I). Clinical skills acquirement was also reduced severely or completely except for the elective rotations since the trainees could rotate in to different medical and surgical specialties according to their laid-down curriculum. E-log book entries were also largely unaffected since residents were keeping a record of the patients (COVID-19) through online portal. Majority of the residents reported non-availability of online learning and assessment (Table-II) and only 59% used telemedicine to decrease the patient load without unnecessary exposure. Increase in duty hours was relayed by 35.2% of the residents. Many of the residents (67-69%) felt complete clinical, educational and psychological abandonment in their departments (Table-II).

**Table-I:** Impact of the pandemic on different aspects of residency.

	*<40% reduction (n%)*	*40-80% reduction (n%)*	*>80% reduction (n%)*
***Teaching aspect of residency***			
MDT meetings	40 (38.1)	26 (24.8)	39 (37.1)
Clinical presentations	31 (29.5)	26 (24.8)	48 (45.7)
Morning meetings	30 (28.6)	27 (25.7)	48 (45.7)
Case based discussions	11 (10.5)	25 (23.8)	69 (65.7)
Grand rounds	26 (24.8)	22 (21)	57 (54.3)
Clinico-pathological conferences	21 (20)	19 (18.1)	65 (61.9)
Teaching sessions by the consultants	16 (15.2)	28 (26.7)	61 (58.1)
Teaching sessions by trainees to juniors	13 (12.4)	21 (20)	71 (67.6)
***Clinical skills***			
Hands-on	7 (6.7)	27 (25.7)	71 (67.6)
Elective rotations	38 (36.2)	31 (29.5)	36 (34.3)
OPD patient inflow	19 (18.1)	57 (54.3)	29 (27.6)
OT/Procedure room inflow	28 (26.7)	56 (53.3)	21 (20)
Elective procedure cancellation	17 (16.2)	38 (36.2)	50 (47.6)
Level of direct/indirect supervision	15 (14.3)	53 (50.5)	37 (35.2)
Delay in acquiring clinical skills	18 (17.1)	42 (40)	45 (42.9)
Delay in mandatory workshops	10 (9.5)	32 (30.5)	63 (60)
***Personal growth and development***			
Delay in acquiring recommendation/experience certificates	15 (14.3)	47 (44.8)	43 (41)
Delay in post graduate exams and assessments	11 (10.5)	60 (57.1)	34 (32.4)
Cancellation of in-person medical conferences	8 (7.6)	57 (54.3)	40 (38.1)
Effect on clinical research	17 (16.2)	50 (47.6)	38 (36.2)
Evaluations and appraisals	21 (20)	38 (36.2)	46 (43.8)
Acquiring CMEs	10 (9.5)	37 (35.2)	58 (55.2)
Maintaining e-log book	31 (29.5)	59 (56.2)	15 (14.3)
Annual leaves	21 (20)	40 (38.1)	44 (41.9)

**Table-II:** Miscellaneous questions regarding impact of the pandemic on residency.

	*Yes (n%)*	*No (n%)*
Alternative to the lack of educational aspect of training	29 (27.6)	76 (72.4)
Online classes	16 (15.2)	89 (84.8)
Online assessments	15 (14.3)	90 (85.7)
Use of telemedicine	62 (59)	43 (41)
Deployment to other wards/centers?	41 (39)	64 (61)
Increase in duty hours?	37 (35.2)	68 (64.8)
Do you feel clinically supported?	38 (36.2)	67 (63.8)
Do you feel educationally supported?	37 (35.2)	68 (64.8)
Do you feel psychologically supported?	36 (34.3)	69 (65.7)

Gender, specialty, number of work days per month and year of residency were all statistically related to substantial decrease in multiple aspects of the residency programs (Table-III). All these variables (except number of work days) were also statistically related to lack of support from the respective departments. On the contrary, the facility of smart learning helped the trainees feel more supported.

**Table-III:** Relation of demographics and variables of smart learning with the impact of the pandemic on several aspects of residency.

*Variables*	*Clinical aspect of residency*	*Teaching aspect*	*Personal growth and development*	*Increase in duty hours*	*Lack of psychological support*	*Lack of clinical support*	*Lack of educational support*
Gender	P=0.04	P=0.008	P=0.008	P=0.369	P <0.001	P<0.001	P <0.001
Specialty	P<0.001	P=0.001	P=0.023	P=0.004	P <0.001	P=0.001	P=0.002
Year of residency	P=0.184	P=0.017	P=0.06	P=0.127	P=0.02	P=0.001	P=0.019
Days/month in hospital	P=0.004	P=0.01	P=0.006	P=0.402	P=0.57	P=0.51	P=0.12
On-line classes	P=0.429	P=0.034	P=0.021	P=0.07	P=0.04	P=0.07	P=0.001
On-line assessments	P=0.512	P=0.191	P=0.546	P=0.62	P=0.001	P=0.001	P=0.001

## DISCUSSION

Healthcare (including medicines, buildings, beds, equipment, vehicles and healthcare staff) being a finite resource is severely affected by the pandemic and the resulting lockdowns. Patients with chronic illnesses, those requiring screening and surveillance and the non-emergency Out Patient follow ups are also among the badly affected population.^10^,^11^ These so called “cold cases” used to be a great learning opportunity for the residents. The patients themselves have also limited their health seeking behaviour out of fear as well as lack of transport and resources.^12^ Late diagnosis, misdiagnosis and considerable emotional trauma have been seen in previous pandemics as well.^13^

Due to the unexpected emergency condition world-wide, community-centred rather than a patient-centred approach has been advocated including social segregation, performing only emergency procedures, telemedicine etc., which might not be regarded ideal by the patients individually but are paramount in view of community safety.^14^ Guidelines have already been laid down to limit unnecessary exposure for the doctors’ and the patients’ safety including deferral of elective procedures and prioritizing life/limb/sight saving procedures.^15^

Many of the post graduate and entrance exams have been rescheduled causing frustration among residents planning for accreditation and higher/foreign qualifications (GMC registration through PLAB or MRCP/MRCS routes, Specialty Certificate Exams (SCE) and local post-graduate exams like FCPS, MCPS and MD/MS).^16^ Many of our residents fell victim to delay in accreditation (90% reporting severe to complete delay) and final assessments (94% reporting severe to complete delay). Unlike the international training programs where relaxation to the overall requirement to get competencies signed-off in lieu of the current circumstances has been formulated already,^17^ no such facilitation has been provided for our residents.

The rational for sequential rotation of the residents is to conserve resources including PPE and to safeguard the heath of the residents, and as a consequence, the health of the patients and the community as a whole.^2^,^18^ Despite clear guidelines, majority of the residents were working more than the designated days with no “cool off” period in our study. Interestingly, many of the residents did not report increase in duty hours in our study as most of them had already been performing 60 plus hours per week with no legislation for work hour limitation.^19^ It is noteworthy that female residents in our study were more prone to clinical, educational and psychological abandonment causing their training programs to be affected tremendously. Similarly, the interventional specialties (Gastroenterology, Cardiology, Dentistry and Surgery) were facing greater disruption.

The Multi-disciplinary team meetings (MDT) were largely unaffected in our survey which might be because of the reason that many departments lack these kind of meetings to begin with. All in-person conferences have been called off around the globe with introduction of online lectures and didactic educational resources being introduced.^20^,^21^ Smart learning has yet to be introduced in many of the teaching hospitals in our country, the importance of which is reflected from the direct relationship of online-classes/assessments and the low level of psychological stress and the feeling of clinical and educational abandonment as shown in Table-III. It was interesting to note that although smart-learning helped in the teaching and personal development (acquiring CME for example) aspects, it didn’t correlate positively with ticking off the clinical skills (such as hands-on) check-list.

Residents from highly specialized fellowship programs have been redeployed to stations like screening facilities, emergency departments and acute medical intakes to supplement the available medical force.^4^ Many of our senior trainees reported greater disruption of training activities and psychological stress due to a decreased confidence level that may come with a reduced specialty-specific case exposure and possibly re-deployment. According to a survey, it will soon be almost impossible to evaluate the residents for their core competencies and the normal laid-out curriculum.^22^ This would lead to further frustration and fear among the residents as their future seems bleak at the expense of these catastrophic conditions.

To our best knowledge this is one of the first multi-centre studies involving multiple specialities to provide insight in to the disruption of the many facets of residency programs. The data was collected from a single province of the country which might not be a true representation of all the residents country-wide owing to logistics and differences in current pandemic situation region wise. Also the socio-economic struggles of the residents were not discussed which could massively affect the psychological stability and as a consequence performance of the trainees.

## CONCLUSION

The current unprecedented condition has disrupted many aspects of life along with the training and assessment of healthcare front-liners. Overall, our study confirmed that the COVID-19 pandemic has substantially affected the clinical skills, teaching and personal growth of many trainees. There is a decrease in exposure to almost all of the aspects of training with no alternative in the form of smart learning provided to many. Clinical, educational and psychological support, although an extremely important part of healthcare staffing and management, has been largely neglected as well.

### Authors’ Contribution:

**LA:** Contributed to the idea, design and drafting of the manuscript, data collection, statistical analysis and literature review.

**MA:** Contributed to data collection of literature review.

**SKHK:** Contributed to data collection.

**SAHK:** Contributed to critical review.

All the authors take equal responsibility for the accuracy and integrity of the work.

## References

[1] [Internet] (2020). Covid.gov.pk.

[2] Warhadpande S, Khaja M, Sabri S (2020). The Impact of COVID-19 on Interventional Radiology Training Programs:What You Need to Know. Acad Radiol.

[3] ACGME Response to the Coronavirus (COVID-19) [Internet] (2020). ACGME.

[4] Potts J (2020). Residency and Fellowship Program Accreditation:Effects of the Novel Coronavirus (COVID-19) Pandemic. J Am Coll Surg.

[5] COVID-19:Recommendations for Management of Elective Surgical Procedures [Internet] (2020). American College of Surgeons.

[6] Coronavirus Disease 2019 (COVID-19) –Prevention &Treatment [Internet] (2020). Centers for Disease Control and Prevention.

[7] Coe T, Jogerst K, Sell N, Cassidy D, Eurboonyanun C, Gee D (2020). Practical Techniques to Adapt Surgical Resident Education to the COVID-19 Era. Ann Surg.

[8] Koh D (2020). Occupational risks for COVID-19 infection. Occup Med.

[9] Create Free Online Surveys. Quizzes and Forms - FreeOnlineSurveys [Internet] (2020). FreeOnlineSurveys.

[10] Daniele A, Francesco C, Giovanni EC, Francesco E, Cristian F, Giovanni L, Sergio S (2020). The impact of COVID-19 pandemic on Urology residency in Italy. Minerva Urol Nefrol.

[11] Letters to the Editor:Non-COVID patients are skipping ER visits. That can be dangerous [Internet] (2020). Los Angeles Times.

[12] Masroor S (2020). Collateral damage of COVID-19 pandemic:Delayed medical care. J Card Surg.

[13] Moody W, Loudon M, Watkin R, Steeds R, Prendergast B (2011). Infective endocarditis:diagnosis delayed during swine flu pandemic. Postgrad Med J.

[14] Naspro R, Da Pozzo LF (2020). Urology in the time of corona. Nat Rev Urol.

[15] Adhi M (2020). Novel Coronavirus Disease Pandemic and Ophthalmologists Perspectives. J Pak Med Assoc.

[16] Application guidelines for early specialization in interventional radiology (ESIR) [Internet] Accreditation Council of Graduate Medical Education (ACGME).

[17] Special Communication to Diagnostic Radiology Residents. Interventional Radiology Residents Subspecialty Radiology Fellows and Program Directors) [Internet] Accreditation Council of Graduate Medical Education (ACGME).

[18] Backer JA, Klinkenberg D, Wallinga J (2020). Incubation period of 2019 novel coronavirus (2019-nCoV) infections among travellers from Wuhan China, 20-28 January 2020. Euro Surveill.

[19] Working time and breaks |Advice guides |Royal College of Nursing [Internet] (2020). The Royal College of Nursing.

[20] Critical Care Introductory Course (2020). SIR resident fellow student (RFS) section [Internet].

[21] American Board of Surgery COVID-19 Updates [Internet] (2020). Absurgery.org.

[22] UPDATED:Coronavirus (COVID-19) and ACGME Site Visits. Educational Activities and Other Meetings [Internet] (2020). ACGME.

